# BCL2-Associated Transcription Factor 1 Promotes SRC/Hypoxia-Inducible Factor 1 Subunit α-Mediated Cancer Stemness in Radioresistant Triple-Negative Breast Cancer

**DOI:** 10.32604/or.2026.080978

**Published:** 2026-06-16

**Authors:** Yu-Hao Huang, Hao-Yeh Chen, Peng-Ju Chien, Chun-Yu Chen, Shao-Ti Li, Hsueh-Te Lee, Yueh-Chun Lee, Wen-Wei Chang

**Affiliations:** 1 Department of Biomedical Sciences, Chung Shan Medical University, Taichung, Taiwan; 2 Department of Emergency Medicine, Tungs’ Taichung MetroHarbor Hospital, Taichung, Taiwan; 3 Post-Baccalaureate Medicine, National Chung Hsing University, Taichung, Taiwan; 4 Department of Radiation Oncology, Chung Shan Medical University Hospital, Taichung, Taiwan; 5 Institute of Anatomy & Cell Biology, College of Medicine, National Yang Ming Chiao Tung University, Taipei, Taiwan; 6 School of Medicine, Chung Shan Medical University, Taichung, Taiwan; 7 Department of Medical Research, Chung Shan Medical University Hospital, Taichung, Taiwan

**Keywords:** B-cell lymphoma 2 (BCL2) associated transcription factor 1, SRC kinase, hypoxia-inducing factor 1subunit α, triple negative breast cancer, cancer stemness, radiation response

## Abstract

***Backgrounds:*** Triple-negative breast cancer (TNBC) is highly aggressive, insensitive to radiotherapy, and exhibits increased cancer stem cell (CSC) properties, contributing to poor patient outcomes. B-cell lymphoma 2 (BCL2) associated transcription factor 1 (*BCLAF1*) is an oncogene in certain cancers, but its role in TNBC is unclear. This study investigated *BCLAF1*’s involvement in radioresistance and CSC activity in TNBC. ***Methods:*** BCLAF1 expression and clinical significance were analyzed using The Cancer Genome Atlas (TCGA) breast cancer dataset. Radioresistant MDA-MB-231 cells were used to examine BCLAF1’s function. Proto-oncogene SRC (SRC) overexpression, *BCLAF1* knockdown, dasatinib treatment, and hypoxia inducible factor 1 subunit α (HIF-1α) inhibition were employed to elucidate regulatory mechanisms. CSC activity was assessed using tumorsphere formation assays. ***Results:*** Elevated BCLAF1 mRNA levels were associated with advanced pathological and T stages (analysis of variance [ANOVA], *p* = 1.4 × 10^−3^) and poorer overall survival by Kaplan–Meier analysis (*p* = 0.021). BCLAF1 expression was positively correlated with SRC signaling pathway-associated genes, including Kirsten rat sarcoma viral oncogene homolog (*KRAS*), GTPase-activating protein-binding protein 1 (*G3BP1*), and phosphoinositide-3-kinase regulatory subunit 1 (*PIK3R1*). Radioresistant cells exhibited higher BCLAF1 expression. SRC overexpression reduced radiosensitivity, while increasing BCLAF1 levels. *BCLAF1* knockdown suppressed tumorsphere formation. Dasatinib decreased BCLAF1, HIF-1α, and stemness proteins, including octamer-binding transcription factor 4 (OCT-4), Notch intracellular domain (NICD), and cellular myelocytomatosis oncogene (c-Myc). *BCLAF1* knockdown diminished nuclear HIF-1α, and HIF-1α inhibition abrogated BCLAF1-induced tumorsphere formation. ***Conclusions:*** BCLAF1 enhances radioresistance and CSC properties in TNBC via SRC-HIF-1α signaling, suggesting that BCLAF1 is a potential therapeutic target to overcome radioresistance in TNBC.

## 1 Introduction

Breast cancer is one of the most prevalent cancers among women worldwide. In 2022, there were approximately 2.3 million new cases and 670,000 deaths globally [1]. In the US, 316,950 new cases of invasive breast cancer are estimated to occur by 2025 [2]. While mastectomy or breast-conserving surgery can improve prognosis for early-stage disease, mortality remains high upon relapse [3]. Thus, precise therapeutics are essential for breast cancer management.

Triple-negative breast cancer (TNBC), which is characterized by estrogen receptor (ER), progesterone receptor (PR), and human epidermal growth factor receptor 2 (HER2) negativity [4], is often resistant to clinical therapies [5]. According to a meta-analysis by the Early Breast Cancer Trialists’ Collaborative Group [6], radiotherapy reduces the risk of locoregional recurrence within 10 years after breast-conserving surgery from ~25% to 8%, with recent updates confirming sustained reductions over 30 years [7]. Nevertheless, 10-year recurrence rates remain at 4%–13% after radiotherapy [8], influenced by factors such as young age (under 40) [9], ER/PR negativity [10], HER2 positivity [11], TNBC subtype [10], and advanced stage [12]. TNBC shows a propensity for heightened locoregional recurrence following neoadjuvant chemotherapy, surgery, and radiotherapy [4], indicating augmented radioresistance, which in TNBC is closely linked to cancer stem cell (CSC) survival and enrichment after radiotherapy. Indeed, radioresistant TNBC subpopulations, such as the MDA-MB-231-derived radioresistant subline (231-RR), exhibit markedly enhanced CSC properties, including increased sphere-forming capacity and stemness marker expression, compared to their parental cells [13]. However, the specific molecular networks that enable these CSC subpopulations to orchestrate radiation survival, sustain stemness, and drive tumor recurrence in TNBC are poorly understood and are a critical knowledge gap in the field.

BCL2-associated transcription factor 1 (BCLAF1) is a nuclear protein initially identified as a transcriptional repressor that promotes death and interacts with the adenoviral Bcl-2 homolog E1B19K [14]. It plays multifaceted roles in cellular processes. Under normal conditions, BCLAF1 is upregulated at apoptosis initiation and downregulated upon BCL2 elevation. Knockout of *BCLAF1* in mice is embryonic lethal due to lung developmental defects [15]. As a transcriptional regulator, BCLAF1 binds to DNA via homologies with the basic leucine zipper (bZIP) and Myb domains [16]. BCLAF1 is implicated in the DNA damage response (DDR), where it promotes TP53 transcription via interaction with protein kinase δ (PKCδ) [17], and is associated with phosphorylated histone H2AX at serine 139 (γ-H2AX) following ionizing radiation, thereby regulating apoptosis and DNA repair [18]. In parallel, evidence increasingly implicates BCLAF1 in the DDR and radioresistance. In addition to its known interaction with γ-H2AX, BCLAF1 facilitates the selective mRNA splicing and nuclear export of key DDR components, such as ATM (ataxia-telangiectasia, mutated) [19]. Moreover, BCLAF1 phosphorylation has been shown to enhance DNA repair efficiency and confer radioresistance in gastric cancer [20]. Given that enhanced DNA repair capacity is a hallmark of radioresistant cancer cells, these findings collectively position BCLAF1 as an important regulator of cellular survival following ionizing radiation.

Beyond its involvement in the DDR, the overarching role of BCLAF1 in cancer biology is complex and highly context-dependent. BCLAF1 repression hinders acute myeloid leukemia progression by arresting cells in the pre-G1 phase and enhancing the efficacy of chemotherapy [21]. BCLAF1 also contributes to apoptosis and autophagic cell death in cancers such as multiple myeloma, suggesting a tumor-suppressive function [14,22]. However, BCLAF1 promotes angiogenesis in hepatocellular carcinoma by regulating hypoxia-inducible factor 1-α (HIF-1α) transcription [23] and drives bladder cancer progression by activating SET and MYND domain-containing protein 3 (SMYD3)-mediated autophagy [24], indicating its oncogenic potential. In breast cancer, BCLAF1 has been shown to act as a key effector in doxorubicin-induced senescence [25]. Our previous study showed that BCLAF1 was upregulated in radioresistant TNBC cells [26]. Despite these advances, the precise mechanisms by which BCLAF1 contributes to radiation resistance and CSC maintenance specifically in TNBC have yet to be fully elucidated. To address this gap, we sought to identify upstream regulators and downstream effectors of BCLAF1 under cellular stress conditions. Building on the evidence that SRC kinase acts as a central driver of therapeutic resistance and pro-survival signaling in breast cancer [27], and that HIF-1α serves as a master regulator of the CSC phenotype and hypoxia adaptation [28], we hypothesized that an interconnected signaling axis involving SRC, BCLAF1, and HIF-1α may act in coordination to regulate the radioresistant and stem-like properties of TNBC.

This study aimed to investigate the role of BCLAF1 in the radiation response and cancer stemness of TNBC, with the goal of assessing its potential as a therapeutic target to enhance radiosensitivity and overcome treatment resistance. To this end, the TCGA database was used to evaluate the clinical value of BCLAF1 mRNA levels and determine the potential involvement of SRC proto-oncogene (SRC) non-receptor tyrosine kinase. The impact of BCLAF1 on radiosensitivity and cancer stem cell activity was assessed using MDA-MB-231 cells and the derived radioresistant cells. Manipulations involving SRC and HIF-1α, such as overexpression and the use of small-molecule inhibitors, were employed to shed light on the signaling axis of BCLAF1’s oncogenic function in TNBC cells.

## 2 Materials and Methods

### 2.1 Cell Line and Culture Conditions

The TNBC cell line, MDA-MB-231 (referred to as 231-P), was purchased from Bioresource Collection and Research Center (BCRC, Hsinchu City, Taiwan; cat. No. 60425, Lot No. 02140). HEK-293T cell line was purchased from the Japanese Collection of Research Bioresources (JCRB) Cell Bank (Osaka, Japan; cat. No. JCRB9068, Lot. No. 08202010). All the cells were cultured in Dulbecco’s modified Eagle’s medium (DMEM; cat. no. 11965092; Gibco™, Thermo Fisher Scientific, Waltham, MA, USA) supplemented with 10% fetal bovine serum (cat. no. SH30071; HyClone, Logan, UT, USA), 1 mM sodium pyruvate (cat. no. 11360070; Gibco™, Thermo Fisher Scientific, Waltham, MA, USA), 2 mM L-glutamine (cat. no. 25030081; Gibco™, Thermo Fisher Scientific, Waltham, MA, USA), and 100 μg/mL of antibiotics (penicillin/streptomycin/amphotericin B; cat. no. 03-033-1B; Biological Industries, Beit-Haemek, Israel). Cells were cultured in accordance with ATCC guidelines. Cell line authenticity was verified via short tandem repeat profiling at the Center for Genomic Medicine, National Cheng Kung University (Tainan, Taiwan). The radioresistant MDA-MB-231 subline (designated as 231-RR) was generated as previously described [26]. To maintain the radioresistant phenotype, 231-RR cells were irradiated weekly with 2 Gy of ionizing radiation using a linear accelerator (Versa HD; Elekta, Stockholm, Sweden). For all cell lines, the absence of mycoplasma contamination was verified using the MycoAlert™ PLUS Mycoplasma Detection Kit (cat. no. LT07-710; Lonza Bioscience, Walkersville, MD, USA).

### 2.2 Chemicals and Antibodies

Dasatinib (cat. no. 11498) and CAY10585 (cat. no. 10012682) were purchased from Cayman Chemical (Ann Arbor, MI, USA). Primary antibodies against p-SRC (Tyr416) (cat. no. AP0480; 1:500) and SRC (cat. no. A19119; 1:1000) were obtained from ABclonal Inc. (Woburn, MA, USA). Antibodies targeting BCLAF1 (cat. no. 26809-1-AP; 1:1000), HIF-1α (cat. no. 20960-1-AP; 1:1000), c-Myc (cat. no. 10828-1-AP; 1:1000), OCT4 (cat. no. 11263-1-AP; 1:1000), and Tubulin (cat. no. 66031-1-Ig; 1:5000) were purchased from Proteintech Group Inc. (Rosemont, IL, USA). Primary antibodies specific to NOTCH1 (cat. no. NB100-78486; 1:1000) were purchased from Novus Biologicals (Centennial, CO, USA). Primary antibodies specific to GAPDH (cat. no. GTX100118; 1:5000), lamin B1 (cat. no. GTX 103292; 1:1000), and tubulin (cat. no. GTX27291; 1:2000) were purchased from GeneTex International Corporation (Hsinchu City, Taiwan). Primary antibodies specific to HA-tag (cat. no. sc-7392; 1:1000) were purchased from Santa Cruz Biotechnology Inc. (Dallas, Texas, USA). Peroxidase-conjugated secondary anti-rabbit IgG (cat. no. 31460; 1:10,000) was purchased from Thermo Fisher Scientific Inc. (Waltham, MA, USA). GAPDH or Tubulin was used as an internal control.

### 2.3 Colony Formation Assay

231-RR cells were seeded at a density of 200 cells per well in 12-well plates and incubated at 37°C for 7–10 days to allow for colony formation. The colonies were then fixed with 2% formaldehyde at room temperature for 5 min and stained with 1% crystal violet (cat. no. 32675; MilliporeSigma, Burlington, MA, USA) for 1 h. Colonies containing ≥50 cells were visualized using an inverted light microscope (AE30; Motic, Hong Kong) and manually counted.

### 2.4 Tumorsphere Formation Assay

Tumorsphere formation assays were performed as previously described [29]. Briefly, single-cell suspensions of MDA-MB-231 and 231RR (radioresistant subline derived from MDA-MB-231) were cultured in DMEM/F12 medium (cat. no. 11320033; Gibco™, Thermo Fisher Scientific, Waltham, MA, USA) supplemented with 0.4% BSA (cat. no. A3311; MilliporeSigma, Burlington, MA, USA), 1× serum-free B27 supplement (cat. no. 17504-044; Gibco™, Thermo Fisher Scientific, Waltham, MA, USA), 20 ng/mL EGF (cat. no. AF-100-15; PeproTech, Cranbury, NJ, USA), 20 ng/mL basic FGF (cat. no. 100-18B; PeproTech), 5 μg/mL insulin (cat. no. I5500; MilliporeSigma), 1 μg/mL hydrocortisone (cat. no. H0135; MilliporeSigma), and 4 μg/mL heparin (cat. no. H0878; MilliporeSigma, Burlington, MA, USA). Cells (1 × 10^4^ per well) were seeded into ultra-low attachment 6-well plates (Greiner Bio-One, Kremsmünster, Austria) and incubated at 37°C in a humidified atmosphere containing 5% CO_2_ for 7–10 days to allow tumorsphere formation. All tumorsphere formation assays were performed as four independent biological replicates, and the data are presented as the mean ± standard deviation (SD) of these replicates. Tumorspheres were defined as spherical cellular aggregates with a diameter greater than 50 μm.

### 2.5 Lentiviral shRNA-Mediated Knockdown of BCLAF1

The packaging plasmids pCMVΔ8.91 and pMD.G, along with gene-specific shRNAs targeting BCLAF1 (TRCN0000336704) and LacZ (control; TRCN0000231722), were obtained from the National RNAi Core Facility (Academia Sinica, Taipei, Taiwan). Lentiviral particles were generated by co-transfecting HEK-293T cells with a plasmid mixture containing shRNA (2.5 μg), pCMVΔ8.91 (2.25 μg), and pMD.G (0.25 μg) using NTRII DNA transfection reagent (cat. no. #JT97-N002M; T-Pro Biotechnology, New Taipei City, Taiwan). Viral supernatants were harvested 48 h after transfection, filtered through a 0.45-μm filter, and used to infect target cells at 30% confluence in the presence of 8 μg/mL polybrene. After 24 h of infection, the medium was replaced with fresh culture medium supplemented with 2 μg/mL puromycin to select for stably transduced cells.

### 2.6 Western Blot Analysis

Whole cell lysates were prepared using Nuclear and cytoplasmic extraction (NETN) buffer (100 mM NaCl, 20 mM Tris-Cl [pH 8.0], 0.5 mM Ethylenediaminetetraacetic acid tetrasodium salt dihydrate (EDTA), 0.5% [v/v] NP-40). Total protein (25 μg per lane) was resolved by 10% Sodium dodecyl-sulfate polyacrylamide gel electrophoresis (SDS-PAGE) and subsequently transferred onto 0.45 μm Polyvinylidene fluoride (PVDF) membranes (cat. no. 66547; Pall Corporation, Washington, NY, USA). Membranes were blocked with 5% (w/v) nonfat dry milk in TRIS-buffered saline (TBS) (cat. no. GTX48889; GeneTex, Inc.) for 1 h at room temperature and incubated with primary antibodies at 4°C overnight. Following three washes with TBS containing 0.1% Tween-20 (TBST), the membranes were incubated with horseradish peroxidase (HRP)-conjugated secondary antibodies for 1 h at room temperature. Protein signals were visualized using Pierce ECL Western Blotting Substrate (Thermo Fisher Scientific, Waltham, MA, USA) and imaged with the Amersham Imager 680 (Cytiva, Marlborough, MA, USA).

### 2.7 Analysis of BCLAF1 Expression across Pathological Stages

We used the OncoDB online database (https://oncodb.org/index.html) to evaluate the correlation between BCLAF1 expression and the clinicopathological features of breast cancer [30]. Specifically, we compared *BCLAF1* mRNA expression levels between different pathological T stages (tumor size) and pathological N stages (lymph node involvement). The distribution of gene expression in each stage was visualized using box plots. Statistical differences between multiple-stage groups were assessed using one-way analysis of variance (ANOVA). A *p*-value of < 0.05 was considered statistically significant.

### 2.8 Gene Set Enrichment Analysis

To investigate the potential association between *BCLAF1* expression and specific oncogenic pathways, RNA-seq transcriptomic data from 1212 breast cancer patients were retrieved from The Cancer Genome Atlas (TCGA; https://portal.gdc.cancer.gov). Patients were stratified into two cohorts, a *BCLAF1*-high group (n = 606) and a *BCLAF1*-low group (n = 606), based on *BCLAF1* mRNA abundance, using the median RSEM-normalized expression value as the cutoff. Gene set enrichment analysis (GSEA; GSEA software version 4.3.2) was then performed to identify biological pathways significantly enriched in the *BCLAF1*-high phenotype. The analysis specifically targeted oncogenic signatures from the Molecular Signatures Database (MSigDB, version 2025.1.Hs), including the “BILD_SRC_ONCOGENIC_SIGNATURE”, “HALLMARK_TGF_BETA_SIGNALING”, and “BILD_CTNNB1_ONCOGENIC_SIGNATURE” gene sets. Statistical significance was assessed using the normalized enrichment score (NES), nominal *p*-value, and false discovery rate (FDR). Gene sets with a nominal *p*-value of < 0.05 and an FDR of < 0.25 were considered statistically significant.

### 2.9 Gene Signature and Correlation Analysis

Gene signatures associated with DNA repair and stemness were retrieved from the “Reactome” and “Gene Reference Into Function” datasets, respectively, in the Harmonizome database (version 3.0, released November 20, 2024; https://maayanlab.cloud/Harmonizome/) [31]. To investigate the association between these functional signatures and *BCLAF1* expression, correlation analyses were performed using the Gene Expression Profiling Interactive Analysis 2 (GEPIA2) platform [32] on breast cancer cohorts from the TCGA. Specifically, Pearson correlation coefficients were calculated to assess the relationship between *BCLAF1* expression and the designated DNA repair and stemness signatures. Statistical significance was set at *p* < 0.05. Correction for multiple testing was not applied, given the hypothesis-driven nature of the study.

### 2.10 Tissue Microarray and Immunohistochemistry Analysis

Breast cancer tissue microarrays (TMAs) were purchased from TissueArray.com LLC (cat. no. BR20811 containing 104 cases; Derwood, MD, USA) with informed consent confirmation. Slides underwent deparaffinization, rehydration, and antigen retrieval prior to incubation with anti-BCLAF1 (26809-1-AP, 1:100) and anti-HIF-1α (20960-1-AP, 1:100) antibodies. Immunoreactivity was visualized using a standard ABC method with 3,3′-diaminobenzidine (DAB) substrates (cat. no. 4065; DAKO, Santa Clara, CA, USA). Sections were counterstained with hematoxylin and mounted with Permount (cat. no. 06522; Merck, Saint Louis, MO, USA). Images were acquired using the TissueFAXS Plus system (TissueGnostics GmbH, Vienna, Austria). Quantitative analysis of the immunostaining was performed using TissueQuest software (TissueGnostics). Briefly, whole TMA spot images acquired with the TissueFAXS Plus system were imported into the software. The algorithm was configured to automatically segment individual cells based on hematoxylin counterstaining, which served as a nuclear mask. For each segmented cell, the mean optical density (OD) of the DAB signal was measured specifically within the nuclear compartment, where both BCLAF1 and HIF-1α predominantly localize. The final expression score for each TMA spot was calculated as the average of the nuclear DAB OD of all segmented cells in that spot. This approach provided a continuous, objective measure of overall staining intensity. All analyses were performed using identical software settings and parameters for all samples.

### 2.11 Statistical Analysis

Quantitative data are expressed as mean ± standard deviation (SD). Statistical analyses were performed using GraphPad Prism 5.0 (GraphPad Software, San Diego, CA, USA). Differences between two groups were assessed using the unpaired Student’s *t*-test, while comparisons between multiple groups were performed using one-way analysis of variance (ANOVA), followed by Tukey’s post-hoc test. Survival curves were generated using the Kaplan–Meier method and compared using the log-rank test. A *p*-value of < 0.05 was set as the threshold for statistical significance.

## 3 Results

### 3.1 BCLAF1 Is Associated with Poor Clinical Outcomes in Patients with Breast Cancer

**Figure 1:**
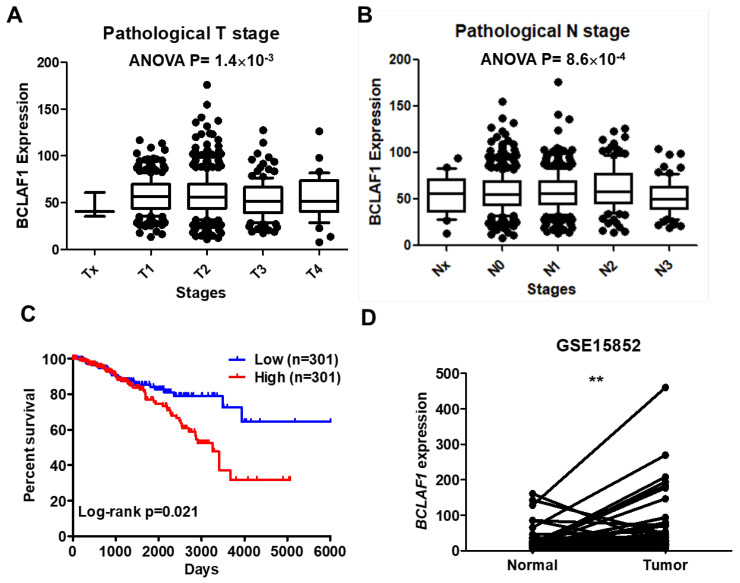
**The prognostic potential of BCLAF1 in breast cancer.** (**A**,**B**) BCLAF1 expression levels across different pathological stages in breast invasive carcinoma samples from The Cancer Genome Atlas (TCGA) dataset retrieved from the OncoDB website. (**A**) BCLAF1 expression by pathological T stage (Tx, T1, T2, T3, and T4). (**B**) BCLAF1 expression by pathological N stage (Nx, N0, N1, N2, and N3). Box plots show medians, interquartile ranges, and individual data points. (**C**) Kaplan–Meier survival curves comparing overall survival between patients with high BCLAF1 expression (top 30%; n = 301; blue line) and low BCLAF1 expression (bottom 30%; n = 301; red line). Survival data were analyzed using the Oncolnc database. (**D**) BCLAF1 mRNA expression levels in paired tumor and adjacent normal tissues from breast cancer patients (n = 43) in the GEO dataset GSE15852. Lines connect matched samples from the same patient. **, *p* < 0.01, paired *t*-test.

To evaluate the clinical relevance of BCLAF1 expression in tumor progression, we analyzed its mRNA expression levels across pathological T and N stages using the OncoDB database [30]. BCLAF1 expression showed significant differences between pathological T stages (ANOVA, *p* = 1.4 × 10^−^^3^; Fig. 1A), with relatively higher expression observed in the T1 and T2 stages compared to TX, although no clear stepwise trend from T1 to T4 was observed. Similarly, significant differences in BCLAF1 expression were observed between pathological N stages (ANOVA, *p* = 8.6 × 10^−^^4^; Fig. 1B). Notably, N2 exhibited a wider interquartile range and higher median expression compared to N3, suggesting that BCLAF1 expression may not be linearly correlated with lymph node metastasis burden. Furthermore, Kaplan–Meier survival analysis of the TCGA breast cancer (TCGA-BRCA) dataset based on the OncoLnc database revealed that the overall survival of patients with high BCLAF1 expression (top 30%; n = 301) was significantly worse compared to that of patients with low expression (bottom 30%; n = 301; *p* = 0.021; Fig. 1C). Additionally, analysis of the GSE15852 dataset revealed that BCLAF1 mRNA levels were upregulated in breast cancer tissues compared to paired adjacent normal tissues (n = 40 pairs; *p* < 0.01; Fig. 1D). Collectively, these findings indicate that elevated BCLAF1 expression is closely associated with tumor progression and poor clinical outcomes in breast cancer.

### 3.2 BCLAF1 Expression Is Correlated with SRC Kinase Signaling in Breast Cancer

**Figure 2:**
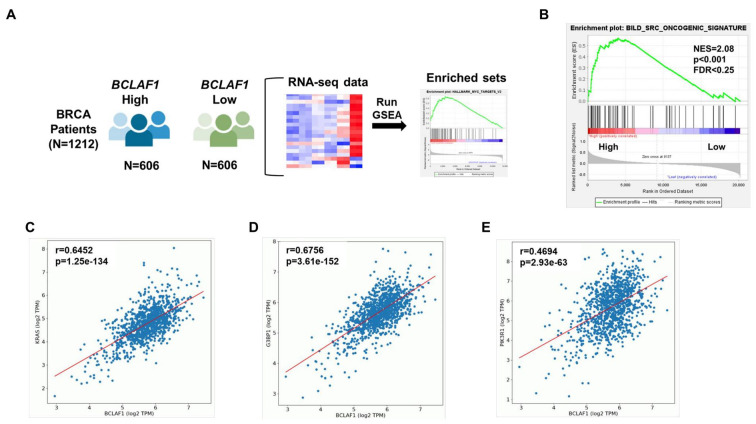
**BCLAF1 expression is correlated with the SRC kinase signaling pathway in breast cancer.** (**A**) Workflow schematic of gene set enrichment analysis (GSEA) performed for The Cancer Genome Atlas breast invasive carcinoma (TCGA-BRCA) cohort stratified by median BCLAF1 expression into high- and low-expression groups. (**B**) GSEA enrichment plot showing the BILD_SRC_ONCOGENIC_SIGNATURE gene set based on BCLAF1 expression levels. The top panel shows the enrichment score curve, the middle panel the ranked gene list with vertical bars indicating genes in the signature, and the bottom panel the ranking metric scores. (**C**–**E**) Scatter plots of correlation between BCLAF1 mRNA expression (log2 TPM) and SRC kinase pathway-related genes in the TCGA-BRCA dataset: (**C**) KRAS, (**D**) G3BP1, and (**E**) PIK3R1. Red lines represent linear regression fits. Each dot represents an individual patient sample.

To investigate the potential signaling pathways associated with BCLAF1 in breast cancer, we performed GAEA using the TCGA-BRCA dataset with the median expression level as a cutoff (Fig. 2A). Our analysis revealed that gene signatures related to TGF-β (Fig. S1A), β-catenin (Fig. S1B), and SRC signaling (Fig. 2B) were significantly enriched in patients with high *BCLAF1* expression. Given that SRC kinase has been reported as a pivotal mediator of the crosstalk between TGF-β and β-catenin signaling [33,34], we further examined the relationship between BCLAF1 and SRC pathway components. Correlation analysis demonstrated significant positive associations between BCLAF1 and SRC pathway-related genes, including Kirsten rat sarcoma viral oncogene homolog (KRAS) (Fig. 2C), GTPase-activating protein-binding protein 1 (G3BP1) (Fig. 2D), and phosphoinositide-3-kinase regulatory subunit 1 (PIK3R1) (Fig. 2E). Taken together, these findings suggest that *BCLAF1* functions as a putative oncogene in breast cancer, potentially through modulation of SRC kinase signaling.

### 3.3 BCLAF1 Promotes Radioresistance in TNBC

**Figure 3:**
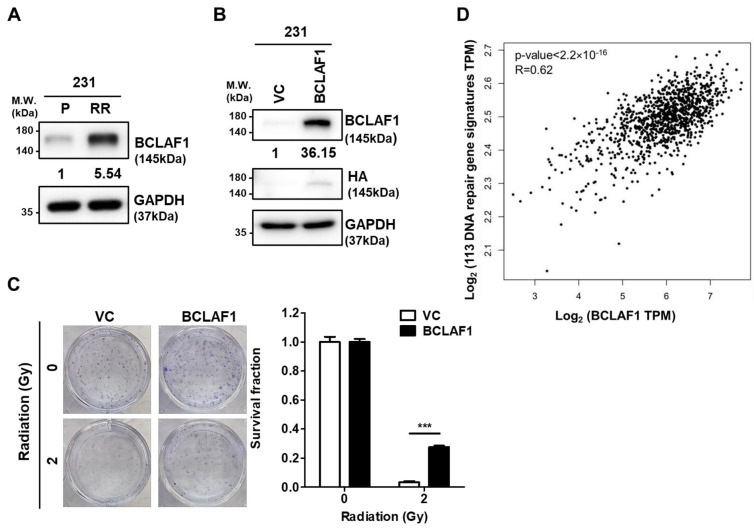
**BCLAF1 contributes to radioresistance in triple-negative breast cancer cells.** (**A**) Western blot analysis comparing BCLAF1 protein expression between parental MDA-MB-231 cells (P) and radioresistant MDA-MB-231 cells (RR) after 6 Gy irradiation. GAPDH serves as the loading control. Molecular weights (kDa) are indicated on the left. (**B**) Western blot confirmation of BCLAF1 overexpression in MDA-MB-231 cells transfected with a BCLAF1 cDNA construct (BCLAF1) or an empty vector control (VC). HA-tag and BCLAF1 expression levels are shown with GAPDH as the loading control. Molecular weights (kDa) are indicated on the left. (**C**) Clonogenic survival assay examining radiation response in BCLAF1-overexpressing MDA-MB-231 cells. The left panel shows representative images of colony formation of vector control (VC) and BCLAF1-overexpressing (BCLAF1) cells after 2 Gy irradiation. The right panel shows quantitative analysis of the survival fraction. Data are shown as mean ± SD. ***, *p* < 0.001. (**D**) Analysis of the correlation between BCLAF1 expression and DNA repair gene signature in the TCGA-BRCA cohort performed using the GEPIA2 platform. Each dot represents an individual patient sample.

Our previous study demonstrated that tribbles pseudokinase 3 (TRIB3) promotes radioresistance in breast cancer cells and identified BCLAF1 as a TRIB3-interacting protein [26]. To investigate whether BCLAF1 also regulates radioresistance in TNBC cells, we first examined its expression and observed elevated BCLAF1 protein levels in radioresistant MDA-MB-231 (231-RR) cells compared to parental cells (Fig. 3A). Next, we overexpressed BCLAF1 in parental MDA-MB-231 (231) cells and confirmed overexpression efficiency using Western blot analysis (Fig. 3B). Clonogenic survival assays demonstrated that BCLAF1 overexpression significantly reduced radiosensitivity in 231 cells (Fig. 3C). Furthermore, analysis of the TCGA-BRCA dataset using the GEPIA2 platform [32] revealed a positive correlation between BCLAF1 expression and DNA repair gene signatures (Fig. 3D; gene lists provided in Supplementary Table S1). Collectively, these findings indicate that BCLAF1 promotes radioresistance in TNBC, potentially by enhancing DNA repair capacity.

### 3.4 BCLAF1 Maintains Cancer Stemness in Radioresistant TNBC Cells

**Figure 4:**
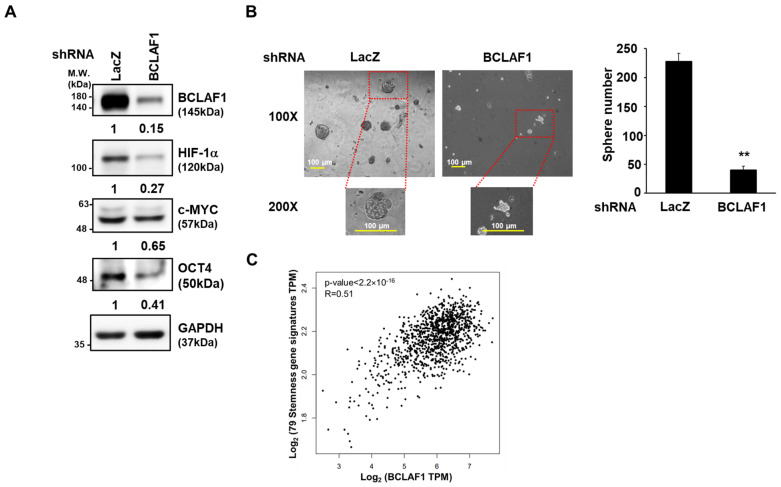
**Knockdown of *BCLAF1* suppresses cancer stemness in triple-negative breast cancer cells.** (**A**) Western blot analysis of stemness-associated proteins in MDA-MB-231 cells transduced with control shRNA (LacZ) or BCLAF1-targeting shRNA (shBCLAF1). Expression levels of BCLAF1, HIF-1α, c-MYC, and OCT4 were examined, with GAPDH serving as the loading control. Molecular weights (kDa) are indicated on the left. (**B**) Mammosphere formation assay comparing control shRNA (LacZ) and BCLAF1 shRNA (shBCLAF1) cells. Left panels show representative bright-field microscopy images of mammospheres. Red dashed boxes indicate the magnified regions shown below. Scale bars: 100 μm. Right panel shows quantification of mammosphere numbers. Data represent mean ± SD. **, *p* < 0.01. (**C**) Analysis of the correlation between BCLAF1 expression and stemness gene signature in the TCGA-BRCA cohort performed using the GEPIA2 platform. Each dot represents an individual patient sample.

We previously reported that 231-RR cells exhibit enhanced cancer stemness compared to 231 cells [13]. To investigate whether BCLAF1 modulates CSC properties in radioresistant TNBC cells, we performed lentiviral shRNA-mediated knockdown of *BCLAF1* in 231-RR cells. BCLAF1 depletion significantly downregulated key CSC-related factors, including HIF-1α, c-MYC, and octamer-binding transcription factor 4 (OCT4) (Fig. 4A). Furthermore, the three-dimensional tumorsphere assay revealed that *BCLAF1* knockdown markedly impaired the sphere-forming ability of 231-RR cells (Fig. 4B). Clinically, a positive correlation between BCLAF1 expression and stemness gene signatures was also identified in the TCGA-BRCA dataset (Fig. 4C; gene lists provided in Supplementary Table S2). Collectively, our data indicate that BCLAF1 plays a positive regulatory role in sustaining the CSC phenotype in radioresistant TNBC cells.

### 3.5 SRC Kinase Upregulates BCLAF1 Expression in TNBC Cells

**Figure 5:**
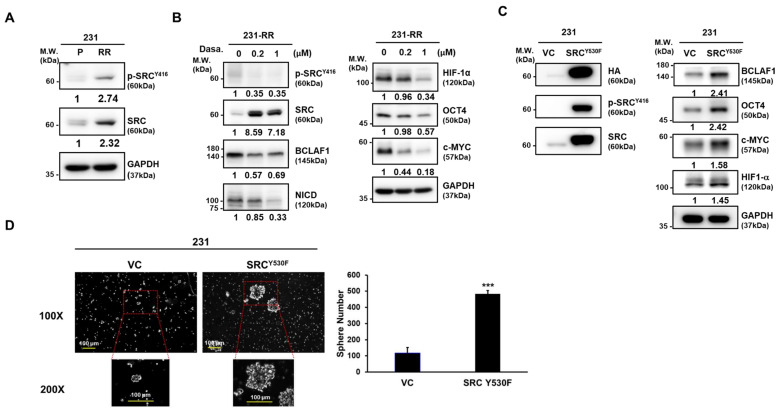
**SRC kinase is an upstream regulator of *BCLAF1* in triple-negative breast cancer cells.** (**A**) Western blot analysis comparing protein expression between parental MDA-MB-231 cells (P) and radioresistant MDA-MB-231 cells (RR). Expression levels of p-SRC^Y416^ and total SRC were examined, with GAPDH serving as the loading control. Molecular weights (kDa) are indicated on the left. (**B**) Dose-dependent effects of dasatinib treatment on protein expression in radioresistant MDA-MB-231 cells (231-RR). Cells were treated with increasing concentrations of dasatinib (0, 0.2, 1 μM) for 48 h. The levels of BCLAF1, p-SRC^Y416^, total SRC, NICD, HIF-1α, OCT4, and c-MYC proteins were detected using Western blot, with GAPDH as the loading control. Molecular weights (kDa) are indicated on the left. (**C**,**D**) Parental MDA-MB-231 cells (231-P) were transfected with an empty vector (VC) or a vector containing a constitutively active SRC mutant (SRC^Y530F^) for 48 h. (**C**) SRC overexpression was confirmed by detecting HA-tag, total SRC, and p-SRC^Y416^. The levels of BCLAF1, OCT4, c-MYC, and HIF-1α proteins were measured using Western blot, with GAPDH as the loading control. Molecular weights (kDa) are indicated on the left. (**D**) Cancer stem cell activity was assessed using tumorsphere formation assay. The left panel shows representative tumorsphere images. Red dashed boxes indicate the magnified regions shown below. Scale bars: 100 μm. Right panel shows quantitative analysis of tumorsphere numbers. Data represent mean ± SD. ***, *p* < 0.001.

To elucidate the regulatory relationship between SRC kinase and BCLAF1 in radioresistant TNBC, we first examined SRC status and observed that both total SRC protein and its phosphorylated form were elevated in 231-RR cells (Fig. 5A). Pharmacological inhibition of SRC activity using dasatinib significantly decreased the expression of BCLAF1 and several key CSC-related factors, including HIF-1α, c-MYC, OCT4, and the Notch intracellular domain (NICD), in 231-RR cells (Fig. 5B). Conversely, to test the effect of SRC activation, we generated 231 cells stably expressing a constitutively active SRC mutant (SRC^Y530F^) [35]. Ectopic expression of active SRC^Y530F^ in 231 cells resulted in elevated BCLAF1 levels and enhanced CSC activity (Fig. 5C,D). Overall, these data suggest that hyperactive SRC signaling drives BCLAF1 overexpression to maintain the stem-like phenotype in radioresistant TNBC cells.

### 3.6 HIF-1α Acts as a Mediator of BCLAF1-Induced Oncogenic Activity in TNBC Cells

**Figure 6:**
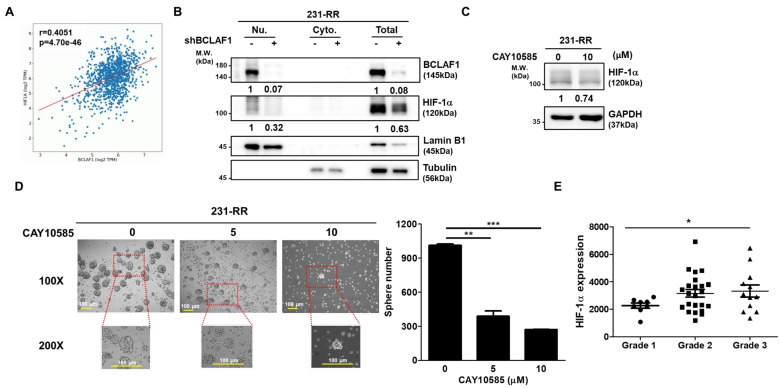
**HIF-1α mediates the oncogenic activity of BCLAF1 in triple-negative breast cancer cells.** (**A**) Scatter plot of correlation between BCLAF1 expression (log2 transcripts per million [TPM]) and HIF-1α expression (log2 TPM) in the TCGA-BRCA cohort. The red line represents the linear regression fit. Each dot represents an individual patient sample. (**B**) Western blot analysis of cytosolic and nuclear fractions from *BCLAF1*-knockdown radioresistant MDA-MB-231 cells (231-RR) after transduction with lentiviruses containing the control shRNA (shLacZ, indicated as “−”) or BCLAF1-specific shRNA (shBCLAF1, indicated as “+”). Tubulin and lamin B1 serve as cytosolic and nuclear loading controls, respectively. Numbers indicate relative expression levels compared to the shLacZ group. Molecular weights (kDa) are indicated on the left. (**C**) Western blot analysis of HIF-1α expression in 231-RR cells treated with vehicle control (0.1% DMSO) or HIF-1α inhibitor CAY10585 (10 μM) for 48 h. GAPDH serves as the loading control. Molecular weights (kDa) are indicated on the left. (**D**) Effect of HIF-1α inhibition on cancer stem cell activity. 231-RR cells were treated with vehicle (DMSO), CAY10585 alone, or BCLAF1 overexpression (BCLAF1) with or without CAY10585, and tumorsphere formation was assessed. Upper panels show representative tumorsphere images. Red dashed boxes indicate the magnified regions shown below. Scale bars: 100 μm. Lower panel shows quantitative analysis of tumorsphere numbers. Data represent mean ± SD from three independent experiments. **, *p* < 0.01; ***, *p* < 0.001. (**E**) HIF-1α expression levels in breast cancer specimens from the GSE15852 dataset, stratified by histological grade (Grade 1, n = 8; Grade 2, n = 23; Grade 3, n = 12). Data are presented as mean ± SEM. *, *p* < 0.05 in one-way ANOVA followed by Tukey’s multiple comparison test.

Although BCLAF1 has been reported to regulate HIF-1α transcription and promote angiogenesis in hepatocellular carcinoma [23], its regulatory relationship with HIF-1α in TNBC is unclear. In our analysis of the TCGA-BRCA dataset using the OncoDB platform [30], we observed a significant positive correlation between BCLAF1 and HIF-1α expression (Fig. 6A), as well as target genes downstream of *HIF-1α* (Fig. S2). Mechanistically, BCLAF1 depletion in 231-RR cells resulted in a marked decrease in nuclear HIF-1α protein levels (Fig. 6B). To determine whether HIF-1α mediates BCLAF1-driven CSC activity, we treated 231-RR cells with the HIF-1α inhibitor CAY10585 (Fig. 6C). Pharmacological blockade of HIF-1α significantly impaired CSC activity in 231-RR cells (Fig. 6D). High histological grade is a hallmark of aggressive breast cancer characterized by undifferentiated cellular states and enriched cancer stemness features [36]. Consistent with this notion, the analysis of the GSE15852 dataset revealed that elevated HIF-1α expression was significantly associated with advanced tumor histological grades (Grades 1–3) (Fig. 6E). Collectively, these findings demonstrate that HIF-1α acts as a key downstream mediator of BCLAF1, linking BCLAF1 to cancer stemness in TNBC.

### 3.7 Positive Correlation between BCLAF1 and HIF-1α Expression in Breast Cancer Specimens

**Figure 7:**
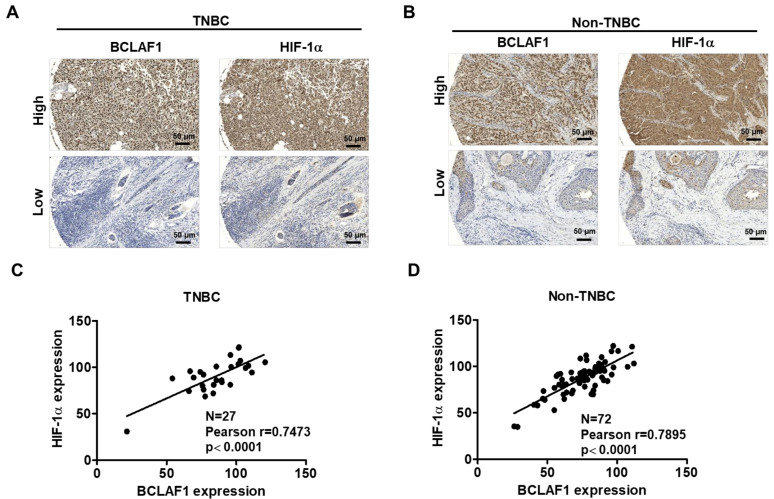
**BCLAF1 expression is positively correlated with HIF-1α levels in breast cancer tissues.** (**A**,**B**) Representative Immunohistochemical (IHC) images showing high (upper panels) and low (lower panels) staining intensities for BCLAF1 (left) and HIF-1α (right) in TNBC (**A**) or non-TNBC (**B**) samples of a breast cancer tissue array. Scale bars: 50 μm. (**C**,**D**) Pearson correlation analysis of scores for BCLAF1 and HIF-1α expression in TNBC (C) or non-TNBC (D) tissues.

To validate these findings in clinical specimens, we analyzed human breast cancer TMAs from 99 cases, including 27 TNBC and 72 non-TNBC patients. Representative immunohistochemical images showing high and low expression levels of BCLAF1 and HIF-1α in TNBC and non-TNBC specimens are presented in Fig. 7A,B, respectively. Consistent with our bioinformatics analyses, significant positive correlations were observed between BCLAF1 and HIF-1α protein levels in both TNBC (Fig. 7C) and non-TNBC tissues (Fig. 7D). Overall, these clinical data provide robust evidence supporting a pathological association between BCLAF1 and HIF-1α in breast cancer.

## 4 Discussion

Triple-negative breast cancer is the most aggressive subtype of breast cancer. It is characterized by early recurrence and a lack of effective targeted therapies [37]. Radioresistance and CSC enrichment are major obstacles contributing to treatment failure and poor patient outcomes [38]. In the present study, we provide evidence that BCLAF1 is associated with radioresistance and cancer stemness in TNBC and may serve as a potential mediator within the SRC/HIF-1α signaling network. We showed that elevated BCLAF1 expression is not only associated with advanced pathological stages and poor prognosis but is also functionally required for maintaining the radioresistant phenotype. Mechanistically, we identified a novel signaling axis wherein hyperactive SRC kinase acts as an upstream driver to upregulate BCLAF1, which subsequently promotes CSC properties through the transcriptional activity of HIF-1α. These findings highlight *BCLAF1* as an oncogene and a promising therapeutic target for sensitizing TNBC to radiotherapy. In addition, the non-linear expression pattern of BCLAF1 across pathological T and N stages (Fig. 1A,B) suggests that its expression is not simply proportional to tumor bulk. Rather, the BCLAF1 level may be modulated by tumor microenvironment heterogeneity and stage-specific regulatory mechanisms. For instance, the relatively elevated BCLAF1 expression observed in early-to-intermediate stages (T1/T2 and N2) might indicate a greater role in facilitating stress adaptation and cancer stem cell maintenance during initial local invasion and regional lymphatic dissemination. In more advanced stages, the development of a complex tumor microenvironment and activation of alternative survival pathways could contribute to the observed plateau or fluctuation in BCLAF1 expression. Further studies are warranted to elucidate the stage-dependent functions of BCLAF1 in TNBC progression. Although our data establish a robust functional link between BCLAF1 and HIF-1α, the precise molecular mechanisms by which BCLAF1 regulates HIF-1α in TNBC warrant further elucidation. Given its well-characterized role as a transcription factor [16], BCLAF1 may primarily act at the transcriptional level. This possibility is supported by the positive correlation between BCLAF1 and HIF1A mRNA expression observed in our TCGA analysis (Fig. 6A), as well as evidence that BCLAF1 directly binds to and activates the HIF1A promoter via its bZIP domain in hepatocellular carcinoma [23]. Nevertheless, we cannot exclude additional post-transcriptional mechanisms or effects on HIF-1α protein stability. For instance, BCLAF1 has been reported to stabilize HIF-1α under hypoxic conditions by reducing its ubiquitination or modulating PHD2 levels in other cancer contexts [39]. Future studies employing chromatin immunoprecipitation assays to confirm promoter binding, along with protein half-life and ubiquitination assays, will be essential to definitively delineate these regulatory interactions and better contextualize the SRC/BCLAF1/HIF-1α axis in radioresistance and cancer stemness of TNBC.

Evidence increasingly highlights the pivotal role of SRC kinase in orchestrating the DDR and conferring radioresistance. Early studies implicated SRC in maintaining cancer cell survival and aggressive phenotypes following cytotoxic stress [40]. Specifically, SRC family kinases have been identified as key regulators in terminating the G2/M DNA damage checkpoint. Kim et al. demonstrated that SRC activity is required to silence the ataxia telangiectasia and Rad3-related protein/checkpoint kinase 1 (ATR/CHK1) signaling pathway following DNA repair, thereby facilitating cell cycle resumption and recovery from genotoxic stress [41]. Consistent with these findings, we observed pharmacological inhibition of SRC markedly increasing the accumulation of γ-H2AX, a marker of DNA double-strand breaks, in irradiated 231-RR cells, indicating that SRC suppression exacerbates radiation-induced DNA damage (data not shown). In the present study, we expand on this regulatory network by identifying BCLAF1 as a critical downstream effector of SRC. Thus, the SRC/BCLAF1/HIF-1α signaling axis represents a comprehensive survival strategy employed by TNBC cells to overcome radiation-induced DNA damage.

The failure of radiotherapy in TNBC is frequently attributed to the inability to eradicate the CSC population [42]. Consequently, the identification of molecular drivers of CSC maintenance is critical for improving therapeutic efficacy. HIF-1α has emerged as a central regulator of breast cancer stem cells. Lu et al. demonstrated that HIF-1α recruits NANOG as a coactivator to induce TERT gene transcription in hypoxic breast cancer stem cells, thereby promoting self-renewal capacity [43]. Additionally, Zhang et al. reported that HIF-1α regulates CD47 expression to facilitate immune evasion and maintenance of the cancer stem cell phenotype [44]. Despite these advances, the upstream regulatory mechanisms governing HIF-1α activation in breast CSCs are incompletely understood. These findings suggest that BCLAF1 acts as a potential regulator that contributes to HIF-1α-associated stemness programs in radioresistant TNBC. We observed that 231-RR cells rely on the SRC/BCLAF1/HIF-1α signaling axis to sustain their sphere-forming capability and elevated expression of cancer stemness factors. Notably, silencing BCLAF1 resulted in diminished nuclear HIF-1α protein levels. Furthermore, silencing BCLAF1 or inhibiting its downstream effector HIF-1α effectively abrogated the CSC phenotype. This finding aligns with a previous study demonstrating that targeting HIF-1α eliminates leukemia stem cells in the xenograft model [45]. These findings suggest that BCLAF1 acts as a potential upstream mediator that activates HIF-1α-associated stemness programs in radioresistant TNBC. Beyond the SRC/BCLAF1/HIF-1α axis, maintenance of the CSC phenotype in TNBC involves complex and interconnected signaling networks. Our GSEA analysis links elevated BCLAF1 expression to TGF-β and β-catenin signatures (Supplementary Fig. S1), while SRC inhibition suppresses Notch signaling components (Fig. 5B). These data suggest that BCLAF1 may participate in crosstalk with several established stemness pathways in TNBC. While our study primarily evaluated stemness through functional tumorsphere assays and core markers (OCT4 and c-MYC), recent evidence highlights FOXA1 and FOXO1 as an important subtype-specific regulators of TNBC stemness [46,47]. The exploration of the potential interplay between the SRC/BCLAF1/HIF-1α axis and these FOXA1/FOXO1 networks is a valuable direction for future investigation. Collectively, these results suggest that current radiotherapy regimens may be insufficient to control TNBC progression unless combined with agents targeting the BCLAF1/HIF-1α network. Disrupting this stemness-sustaining axis offers a promising strategy to resensitize the refractory CSC population to radiation.

The clinical significance of our findings is underscored by the robust association between BCLAF1 expression and poor patient outcomes. Analysis of the TCGA and GEO datasets revealed that elevated BCLAF1 levels are correlated significantly with advanced tumor stages and shorter overall survival. Furthermore, the positive correlation observed between BCLAF1 and HIF-1α in clinical breast cancer specimens reinforces the translational relevance of the SRC/BCLAF1/HIF-1α axis identified in our cellular models. These data suggest that BCLAF1 could serve as a valuable prognostic biomarker for identifying TNBC patients at high risk of radioresistance and recurrence. In future, stratification of patients based on BCLAF1 and SRC activation status may help tailor personalized therapeutic strategies. While our study demonstrates the efficacy of targeting this axis *in vitro*, further *in vivo* studies and clinical trials are warranted to validate the therapeutic benefits of combining SRC or BCLAF1 inhibitors with radiotherapy. In addition to conventional pharmacological and radiation approaches, emerging physical modalities such as cold atmospheric plasma (CAP) have shown promising effects in suppressing TNBC stemness by inhibiting FOXO1 protein levels through promoting its K48-linked ubiquitination [46]. Querying the CAPmed-BC web service with the parameters *p* < 0.05 and |LogFC| > 1.5 (TNBC_Control vs. TNBC cells treated with CAP for 8 h) revealed that SRC, BCLAF1, and HIF-1α were not significantly differentially expressed in the available datasets. Nevertheless, this platform represents a valuable multi-omics resource for exploring CAP responses in breast cancer. Further analysis using CAPmed-BC or larger CAP-treated datasets may help determine whether the modulation of the SRC/BCLAF1/HIF-1α axis contributes to the effects of CAP on TNBC stemness and therapeutic resistance.

While our study demonstrates the efficacy of targeting the SRC/BCLAF1/HIF-1α axis in the MDA-MB-231 cellular model and its radioresistant derivative (231-RR), we acknowledge that the reliance on a single TNBC cell line is a limitation, given the well-documented biological heterogeneity of TNBC [47]. Further validation in additional TNBC cell lines as well as patient-derived models will be necessary to fully account for biological variability and to confirm the generalizability of these findings across different TNBC subtypes. The translation of the SRC/BCLAF1/HIF-1α axis into clinical practice will require careful pharmacokinetic and pharmacodynamic (PK/PD) considerations. Dasatinib, an FDA-approved SRC inhibitor, has been evaluated in breast cancer trials at daily doses of 70–100 mg [48]. However, its combination with radiotherapy needs to be carefully assessed in early-phase clinical trials to establish the maximum tolerated dose and monitor potential overlapping toxicities, such as myelosuppression and fatigue. In contrast, CAY10585 is a preclinical tool compound that, while useful for target validation *in vitro* [49], possesses suboptimal pharmacokinetic properties and potential off-target effects, limiting its direct clinical translation. Therefore, future studies should evaluate more selective, clinically advanced SRC and HIF-1α inhibitors in relevant *in vivo* models to better define their therapeutic window when combined with standard radiotherapy in TNBC.

Furthermore, although our bioinformatic analyses of public datasets (e.g., TCGA) reveal significant positive correlations between BCLAF1 expression and several oncogenic signatures, including SRC signaling, DNA repair, stemness, and HIF-1α target genes, we interpret these findings with caution. We acknowledge that gene expression correlations do not necessarily imply causal or direct functional relationships. These correlative observations primarily serve to generate hypotheses and provide a supportive context for our experimental *in vitro* and mechanistic studies. Additional rigorous functional validation, including targeted perturbation and direct interaction assays, will be essential to conclusively establish any direct regulatory interactions within these networks in TNBC.

In addition, we recognize the inherent limitations of our experimental models. First, the generation of the 231-RR subline through repeated irradiation may induce broader transcriptomic and epigenetic adaptation artifacts beyond the specific pathways investigated in this study. Nevertheless, the core findings on the SRC/BCLAF1/HIF-1α axis were consistently supported by multiple orthogonal approaches, including genetic manipulation of SRC and BCLAF1. Second, although dasatinib and CAY10585 effectively suppressed the activities of SRC and HIF-1α, respectively, we acknowledge that chemical inhibitors inherently carry the risk of off-target effects. To mitigate this concern and confirm on-target specificity, our study employed complementary genetic approaches, including overexpression of a constitutively active SRC mutant (SRC^Y530F^) (Fig. 5C,D) and shRNA-mediated knockdown of *BCLAF1* (Fig. 4A,B). The concordant results obtained from both pharmacological inhibition and genetic interventions strongly support the specific involvement of the SRC/BCLAF1/HIF-1α axis in regulating BCLAF1 expression, cancer stem cell activity, and radioresistance in TNBC.

## 5 Conclusions

In conclusion, our study identifies *BCLAF1* as a novel oncogene that promotes radioresistance and cancer stemness in TNBC. We demonstrate that BCLAF1 expression is elevated in radioresistant TNBC cells and is associated with advanced tumor stages and poor overall survival in breast cancer patients. Mechanistically, we reveal a novel SRC-BCLAF1-HIF-1α signaling axis wherein hyperactive SRC upregulates BCLAF1, which subsequently activates HIF-1α to sustain CSC activity. Targeting this axis, either through *BCLAF1* silencing or pharmacological inhibition of SRC and HIF-1α, effectively suppresses the CSC phenotype in radioresistant cells. In summary, these findings suggest that BCLAF1 may represent a potential prognostic biomarker and a candidate therapeutic target within the SRC/BCLAF1/HIF-1α axis to help overcome radioresistance in TNBC. However, given that our results are predominantly derived from bioinformatic analyses and *in vitro* experiments, further preclinical studies, particularly *in vivo* models, are necessary to validate the underlying mechanisms. Subsequent clinical investigations are required to evaluate the true translational relevance and potential therapeutic benefit of targeting this axis in combination with radiotherapy for TNBC patients.

## Data Availability

The data supporting the findings of this study are available from the corresponding authors upon reasonable request.

## Supplementary Materials

The supplementary material is available online at https://www.techscience.com/doi/10.32604/or.2026.080978/s1.
