# Supplementary material for: BCL2-Associated Transcription Factor 1 Promotes SRC/Hypoxia-Inducible Factor 1 Subunit α-Mediated Cancer Stemness in Radioresistant Triple-Negative Breast Cancer
Source: Oncol Res. 2026 Jun 16;34(7):19. doi: 10.32604/or.2026.080978 (PMC13292053; doi:10.32604/or.2026.080978)
Supplement: Supplementary file 1 [file OncolRes-34-80978-s001.zip › Supplementary_Figures.docx]

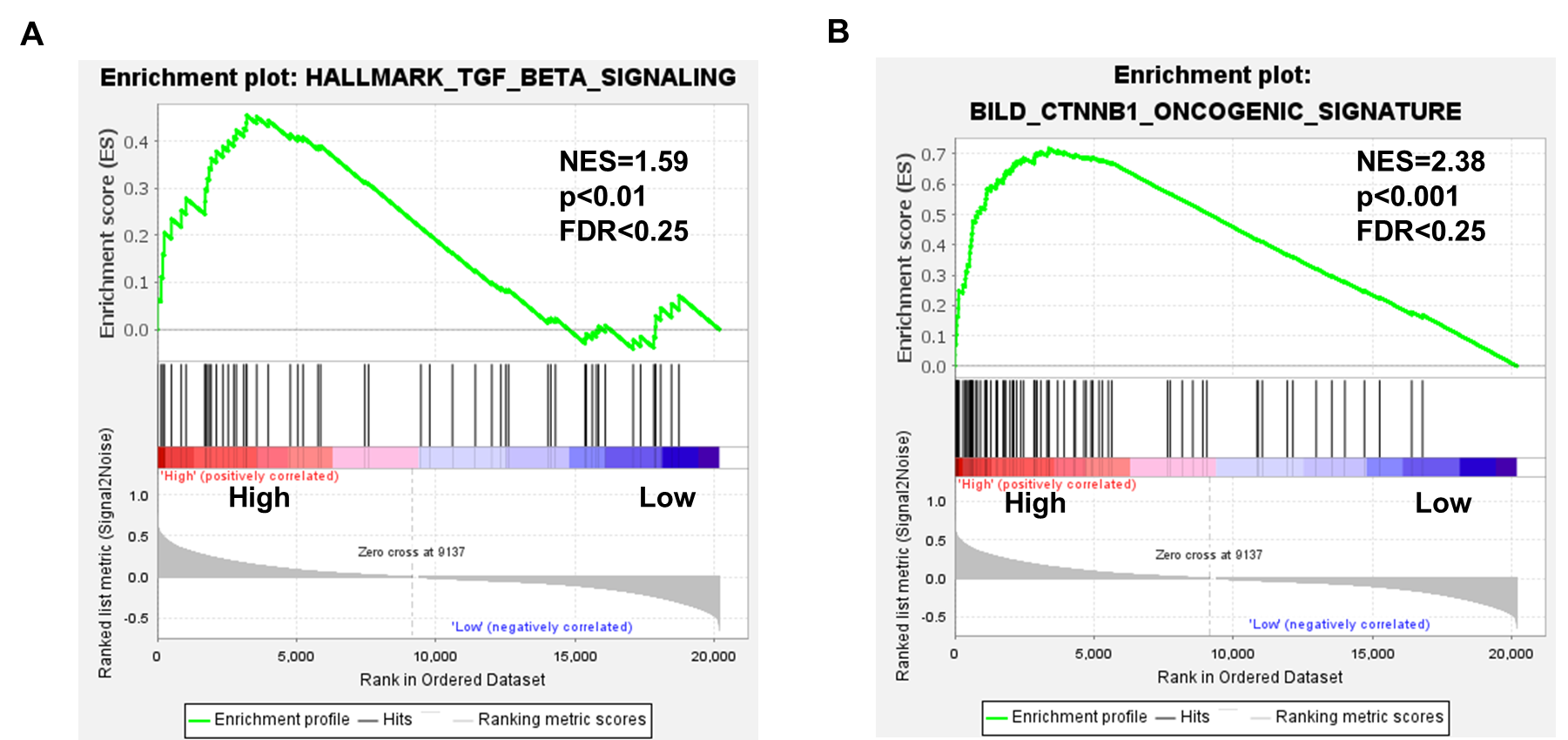


**Figure S1:** Gene set enrichment analysis (GSEA) demonstrates enrichment of oncogenic signatures in BCLAF1-high breast cancer patients. GSEA enrichment plots show significant positive enrichment of (**A**) HALLMARK_TGF_BETA_SIGNALING and (**B**) BILD_CTNNB1_ONCOGENIC_SIGNATURE in breast cancer patients with high BCLAF1 expression from the TCGA-BRCA cohort. The top panel shows the enrichment score curve, the middle panel the ranked gene list with vertical bars indicating genes in the signature, and the bottom panel the ranking metric scores. NES, normalized enrichment score; FDR, false discovery rate.


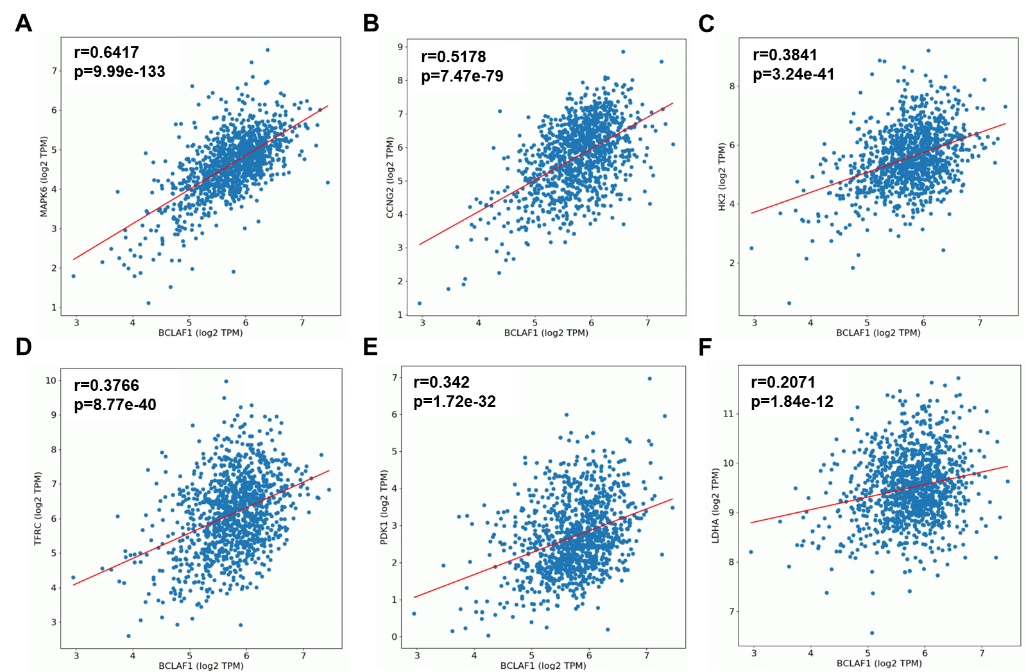


**Figure S2:** BCLAF1 expression is positively correlated with HIF-1α target genes in breast cancer. Scatter plots showing correlations between BCLAF1 expression and HIF1α target genes: (**A**) MAPK6, (**B**) CCNG2, (**C**) HK2, (**D**) TFRC, (**E**) PDK1, and (**F**) LDHA. HIF1α target gene annotations were obtained from the Harmonizome database. Correlation analyses were performed using TCGA-BRCA data via the OncoDB platform. Gene expression values are shown as log2 TPM (transcripts per million). Pearson correlation coefficients (r) and *p*-values are given for each comparison. Red lines represent linear regression fits.
