# Supplementary figures and images for: BCL2-Associated Transcription Factor 1 Promotes SRC/Hypoxia-Inducible Factor 1 Subunit α-Mediated Cancer Stemness in Radioresistant Triple-Negative Breast Cancer
Source: Oncol Res. 2026 Jun 16;34(7):19. doi: 10.32604/or.2026.080978 (PMC13292053; doi:10.32604/or.2026.080978)

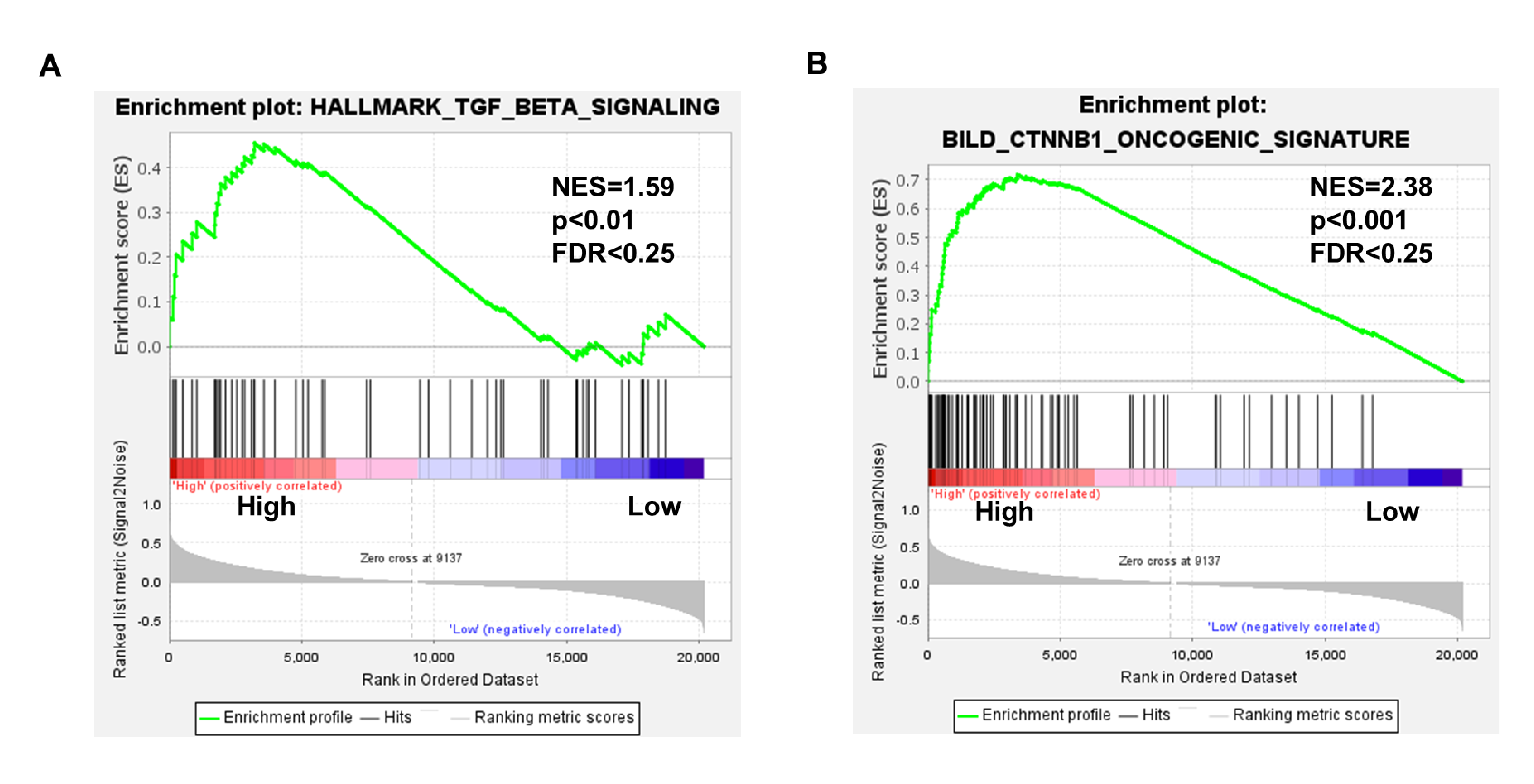

Supplement: Supplementary file 1 [file OncolRes-34-80978-s001.zip › Fig.S1_.tif]
